# Reply: “Comment on: The Vitamin D–Folate Hypothesis as an Evolutionary Model for Skin Pigmentation: An Update and Integration of Current Ideas, *Nutrients* **2018**, *10*, 554”

**DOI:** 10.3390/nu10111759

**Published:** 2018-11-14

**Authors:** Patrice Jones, Mark Lucock, Martin Veysey, Emma Beckett

**Affiliations:** 1 School of Environmental & Life Sciences, University of Newcastle, Ourimbah, NSW 2258, Australia; patrice.jones@uon.edu.au (P.J.); emma.beckett@newcastle.edu.au (E.B.); 2 Hull-York Medical School, University of York, Heslington, York YO10 5DD, UK; martin.veysey@hyms.ac.uk; 3 School of Medicine and Public Health, University of Newcastle, Ourimbah, NSW 2258, Australia

**Keywords:** vitamin D, skin pigmentation, folate, vitamin D-folate hypothesis

## Introduction

We thank Elias and Williams for their interest in our review [1]. While it appears that the authors have taken issue with our focus, we assert that our review takes a balanced approach to integrate current thinking into the prominent evolutionary models for skin pigmentation. In particular, we highlight the fact that this review sought to examine the overlap between these models, rather than aiming to convince the reader of the merits of any one particular hypothesis. 

Contrary to what has been suggested by Elias and Williams, we do not “take issue” with the notion that “pigmentation evolved to optimize permeability and antimicrobial barrier function in the hostile, infectious soup of equatorial Africa”. We remain open-minded about the various hypotheses and present them as such in our manuscript—i.e., as hypotheses, not empirically derived facts. Furthermore, where data is becoming available, it is important to incorporate them into the equation. Elias and Williams’ predilection for discussing congenital abnormalities, while interesting, does not add to the debate. The role of low folate and/or subsequent elevated homocysteine in a great many complications of pregnancy would be more germane to the argument. For example, low folate and/or high homocysteine are associated with lower placental and birth weights (and, hence, perinatal mortality) [2]. Similarly, an aberrant effect on the gene methylation profile would also be likely [3]. Given the intrinsically high rates of miscarriage during very early pregnancy, potential UV-associated folate loss, elevated embryotoxic homocysteine, and specific folate-related gene polymorphisms that alter DNA methyl group-dependent synthesis (depending on folate status) are a nexus of phenomena that would likely act to silently influence embryo survival. Therefore, any argument focusing on a single relationship between a specific congenital anomaly and reproductive success neglects to consider folate (as well as vitamin D) as having extremely complex, polygenic, and pleiotropic actions. 

Elias and Williams’ statement that “folic acid, its metabolites, and its *regulatory genes* can be readily degraded in vitro” is misleading, as the degradation of the genes simply does not occur (notwithstanding thermolability). The authors reference three studies that observe no effects of UVA/B on serum folate levels; however, one of the references provided does not seem related to the points made [4]. Referenced studies by Gambichler et al. (2001) and Juzeniene et al. (2010) report no influence of UVA/B on serum folate levels in small cohorts [5,6], but an equal amount of other studies do report an effect. Notably, Borradale et al. (2014) report that increases in solar UV radiation (UVR) exposure over three weeks reduce the efficacy of folic acid supplements and serum folate levels [7]. Additionally, we have recently shown in a larger population study that surface UV irradiance is associated with long-term systemic folate levels (red cell and serum folate) and that this is strongly influenced by the C677T-*MTHFR* gene variant [8]. Differences in results between studies likely reflect differences in the UVR parameters assessed (UVA or UVB, single dose or cumulative exposures) and whether other parameters of folate are considered in the studies (i.e., red cell folate levels or genetic polymorphism). Further controlled studies are warranted, but a lack of consensus does not represent a lack of evidence. 

In response to Elias and William’s views on vitamin D, we believe their interpretation overlooks several salient points made in our review about the integration of other theories proposed for skin depigmentation and the functions of vitamin D. We mentioned the Metabolic Conservation theory, which proposes that depigmentation involves resources being drawn away from melanin production into other metabolic processes. This theory still supports the likelihood of extreme depigmentation in northern Europeans being a mechanism to facilitate vitamin D production in low-UVR environments. We broadly discussed that the protection of vitamin D and folate status likely acted as a critical selection pressure for skin depigmentation and pigmentation close to the poles and the equator, respectively. These UV-related geophysical factors might drive deficiency in these nutrients, but the primary “driver” in Central European and Asian populations may have been a need to restrict melanin production and channel these resources into responding to increased energy needs associated with colder climates. We make the point that this is not to say that the importance of these nutrients in populations in intermediate UVR environments is undermined—highlighting roles in vasodilation/vasoconstriction and adipocyte biology that would have importance in maintaining energy and temperature homeostasis in such environments. 

Unfortunately, much of the content in our review appears to have been overlooked by Elias and Williams’ in their critique. We present an evidence-based discussion of a likely mutually beneficial relationship between vitamin D, folate, and skin pigmentation that updates and further extends all current theories for the evolution of skin pigmentation and depigmentation. The purpose of our review was not to renounce any of the prominent theories for skin pigmentation and, hence, “cherry-pick” a favourite, but to discuss similarities between them and, hence, potentially extend these models. This purpose appears to have been be missed by Elias and Williams, whose response simply seems to be an argument against the vitamin D–folate hypothesis and a supporting letter for the alternate skin barrier hypothesis. 
